# Borane–Trimethylamine Complex: A Versatile Reagent in Organic Synthesis

**DOI:** 10.3390/molecules29092017

**Published:** 2024-04-27

**Authors:** Dario Perdicchia

**Affiliations:** Dipartimento di Chimica, Università Degli Studi di Milano, Via Golgi 19, 20133 Milan, Italy; dario.perdicchia@unimi.it

**Keywords:** borane, amine–borane complexes, reduction, ionic hydrogenation, radical reactions

## Abstract

Borane–trimethylamine complex (Me_3_N·BH_3_; BTM) is the most stable of the amine–borane complexes that are commercially available, and it is cost-effective. It is a valuable reagent in organic chemistry with applications in the reduction of carbonyl groups and carbon–nitrogen double bond reduction, with considerable examples in the reduction of oximes, hydrazones and azines. The transfer hydrogenation of aromatic *N*-heterocycles and the selective *N*-monomethylation of primary anilines are further examples of recent applications, whereas the reduction of nitrobenzenes to anilines and the reductive deprotection of *N*-tritylamines are useful tools in the organic synthesis. Moreover, BTM is the main reagent in the regioselective cleavage of cyclic acetals, a reaction of great importance for carbohydrate chemistry. Recent innovative applications of BTM, such as CO_2_ utilization as feedstock and radical chemistry by photocatalysis, have extended their usefulness in new reactions. The present review is focused on the applications of borane–trimethylamine complex as a reagent in organic synthesis and has not been covered in previous reviews regarding amine–borane complexes.

## 1. Introduction

Boron-based reagents play an important role in modern organic synthesis and especially borane carriers have reached a predominant position in the synthesis of pharmaceutics and natural products. Boranes form complexes with Lewis bases, such as amines and pyridines, that are stable, safer and easier to handle. There are few reviews in the literature concerning the use of amine–borane complexes in organic synthesis [1,2,3,4,5,6,7,8], but in some of them, the part describing the reactivity is quite limited. An early review [2] is the most representative in the description of the reactivity of several amine–borane complexes with examples of practical application in organic synthesis covering literature up to 1984; it is also the only one that describes some applications of borane–trimethylamine complex (Me_3_N·BH_3_; BTM) while the subsequent reviews report either single reactions with this reagent or deal with different amine–boranes. A review on the chemistry of amine and phosphine–boranes was published [3] in 1999, whereas reductive amination was the topic of two reviews, one focused on amine–boranes [5] and the other with several boron reagents [4]. Amine–boranes forming frustrated Lewis pairs was the subject of a chapter in a book [6], whereas two recent reviews dealt with the reactivity of ammonia-borane complex [7,8]. This review aims to focus on the use of borane–trimethylamine complex as a reagent, mainly after 1984 and not covered in previous reviews regarding amine–borane complexes.

Of the various known complexes, BTM is the most stable [9], less sensitive to hydrolysis, even under acidic conditions [2], and does not require any special storage conditions. It is thermally stable up to 120 °C and can be purified by vacuum sublimation; conversely, ammonia–borane complex explodes when heated, and an attempted distillation of borane–pyridine complex at reduced pressure resulted in violent decomposition [1]. BTM is very soluble in a wide variety of solvents [1], and its stability significantly increases with increasing solvent polarity [10]. It is relatively inexpensive compared with the other amine–borane complexes commercially available and considering the low molecular weight. Contrarily to borane complexes of ammonia, primary and secondary alkylamines, the inert nature of trimethylamine in BTM was a further advantage in avoiding the side reactions observed with the more reactive amines. Its reactivity can be opportunely activated in the reaction medium, and it is considered a valuable reagent in organic synthesis for laboratory as well as industrial scale. Since 1937, when it was synthesized for the first time, its applications have grown steadily, with some innovative ones in recent years.

## 2. Reductive Transformations with Carbon–Oxygen Double Bond

### 2.1. Reduction of Ketones

**Scheme 1:**
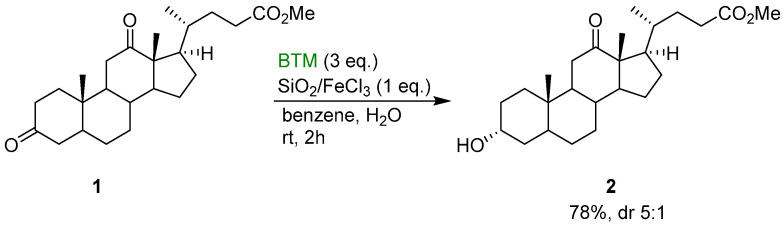
Regioselective reduction of dione **1** on a silica gel surface.

BTM is a weak hydride donor more reactive than trialkylsilanes and trialkylgermanes, comparable with that of trialkylstannanes [11]. The greater stability of BTM in the presence of Lewis or Brønsted acid allows the activation of the substrate by acid catalysis [2]; from kinetic studies, the rate of reduction of aldehydes and ketones increases with increasing acidity of the medium suggesting the formation of a protonated carbonyl species in rapid equilibrium, followed by the rate-determining step of the reduction. BTM was the reagent of choice for the selective reduction of steroidal diones, such as **1**, in the presence of wet silica gel impregnated with FeCl_3_ (Scheme 1): C-3 carbonyl group was reduced preferentially to the alcohol **2** [12].

**Scheme 2:**
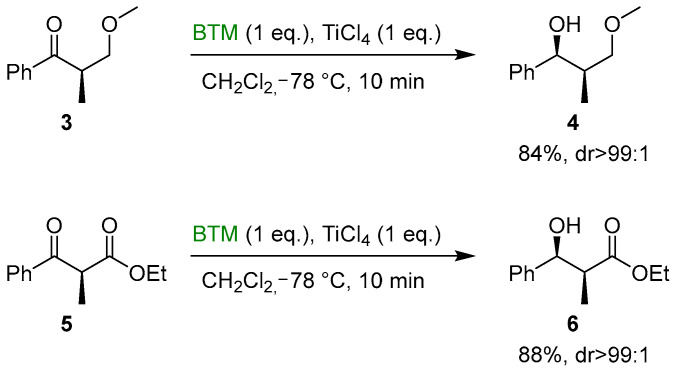
Diastereoselective reduction of ketones **3** and **5**.

The formation of a chelate between the Lewis acid and substrates **3** and **5** proved to be effective for the completely diastereoselective reduction to the corresponding alcohols **4** and **6** (Scheme 2) [13].

**Scheme 3:**
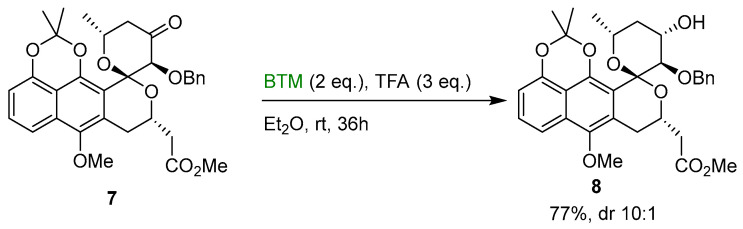
Diastereoselective reduction of ketone **7**.

In a similar way, in the enantioselective total synthesis of analogs of griseusins [14], ketone **7** was reduced by BTM in good diastereoselectivity using TFA as acid (Scheme 3).

### 2.2. Reductive Bromation of Aromatic Carbonyl Compounds

**Scheme 4:**
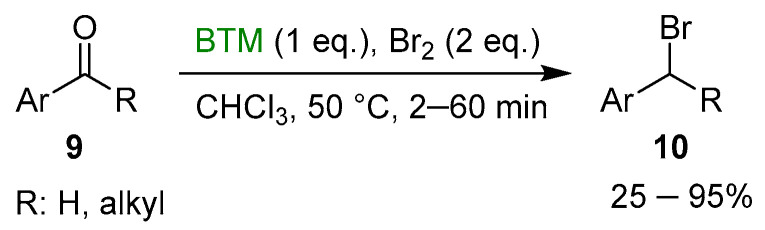
Reductive bromation of aromatic carbonyl compounds.

Bromine was an effective activator in the reduction of both aldehydes and ketones by BTM [15], but in the case of aromatic compounds **9,** the reaction proceeded beyond the reduction to alcohol, leading to bromide derivative **10** in good yields (Scheme 4) with the exception of a product obtained in low yield after recrystallization.

**Scheme 5:**
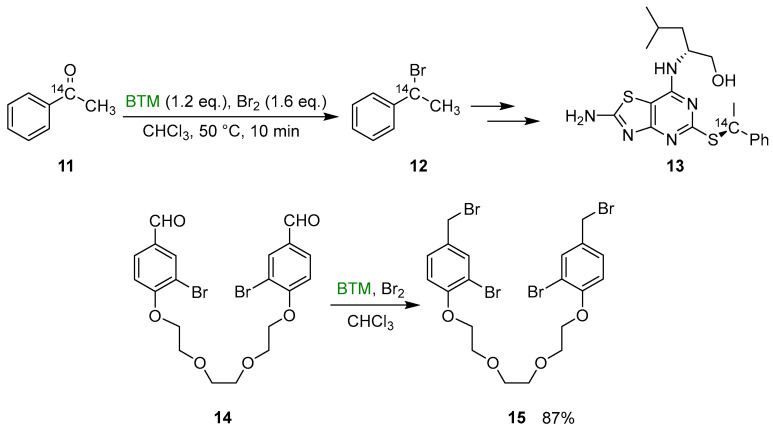
Synthesis of the ^14^C labeled benzylbromide **12** and the dibromide derivative **15**.

Some useful exploitations of the synthetic method were (Scheme 5) the synthesis of the ^14^C labeled benzylbromide **12**, intermediate in the synthesis of CX_3_CR1 antagonist **13** [16], and the synthesis of the dibromide derivative **15**, intermediate in the synthesis of crownophanes [17].

### 2.3. Reduction of Carboxylic Acids

**Scheme 6:**
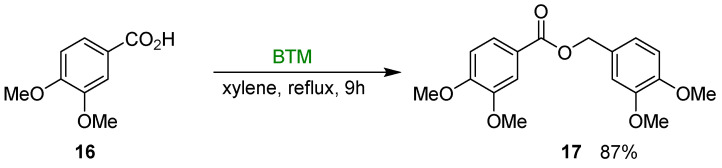
Reduction of acid **16** to ester **17**.

At room temperature, carboxylic acids are inert in presence of BTM and can be used as solvent in the reduction reactions; on the other hand, refluxing a xylene solution of BTM and carboxylic acid **16** in molar ratio of 1.5:2, ester **17** was isolated in 87% yield [18] (Scheme 6); the reaction was suitable both to aliphatic and aromatic acids with moderate to good yields (28–87%).

**Scheme 7:**
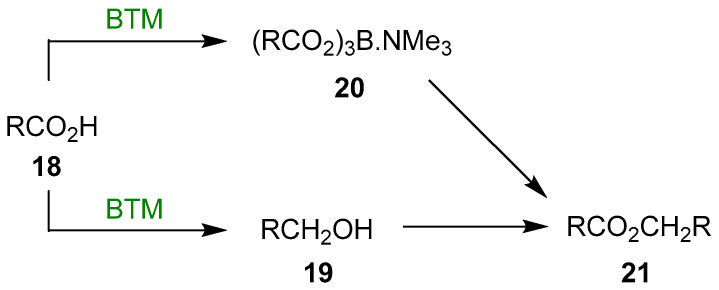
Reaction mechanism of the acid reduction.

A plausible reaction mechanism could be the presence of two concurrent reactions: the reduction of the acid **18** to alcohol **19**, isolated in every reaction, and the formation of the triacyloxyborane complex **20**, a known acylating compound; finally, the reaction of the two intermediates leads to the ester **21** (Scheme 7).

**Scheme 8:**
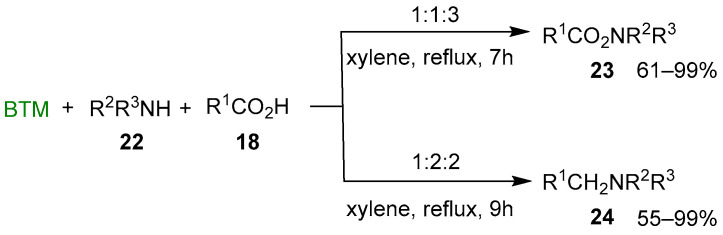
Reaction of *N*-acylation or *N*-alkylation of amines **22** by carboxylic acids **18**.

By adding either primary or secondary amine **22** to the previous reaction mixture and changing the ratio of the reagents, a different result was obtained (Scheme 8) [19]: with the molar ratio BTM:amine **22**:acid **18** = 1:1:3 led to amide **23**, seemingly by the action of acylating complex **20**; instead, the molar ratio 1:2:2 led to the tertiary amine **24**, likely by reduction of the acid **18** to an aldehyde equivalent (boryl acetal) followed by the reductive alkylation of amine **22**.

### 2.4. Reductive Methylation with CO_2_

**Scheme 9:**
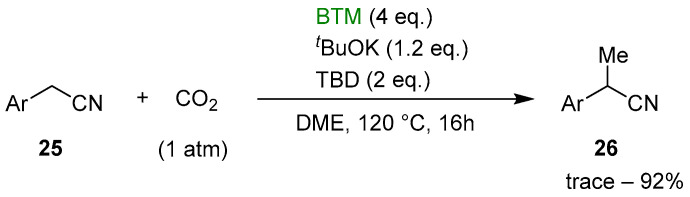
Selective monomethylation of 2-arylacetonitriles **25**.

Recently, the reduction of CO_2_ has emerged as a topic of great interest connected with global climate change and the urgent necessity to reduce the concentration of CO_2_ in the atmosphere through sequestration and its utilization in the synthesis of useful compounds. An early study [20] reported the reduction of CO_2_ to formate by BTM weakly bonded to the bulkier Lewis acid Al(C_6_F_5_)_3_; then, the reaction mechanism was studied by quantum chemical calculations [21]. Subsequently, the reduction of CO_2_ was exploited for the selective monomethylation of 2-arylacetonitriles **25** [22]; as shown in Scheme 9, the optimized reaction conditions involved the reaction, in a sealed tube, of a DME solution of nitrile **25**, *^t^*BuOK and 1,5,7-triazabicyclo[4.4.0]dec-5-ene (TBD) in an atmosphere of CO_2_, obtaining selectively the monomethyl derivative **26**, with yields around 80% in almost all examples.

**Scheme 10:**
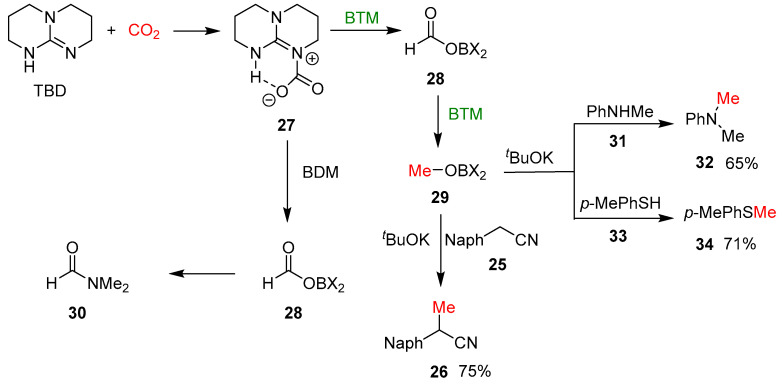
The proposed mechanism of the reaction of monomethylation.

By using ^13^CO_2,_ the corresponding ^13^C methyl derivative was obtained, confirming that the methyl group comes from CO_2_. In the reaction, TBD has the role of activator of the CO_2_ forming the adduct **27** (Scheme 10), and *^t^*BuOK of a strong base in deprotonation of 2-arylacetonitriles **25**. The reaction, shown in Scheme 9, performed without nitrile **25**, led to methyl borate **29** as the major product, which suggests that CO_2_ undergoes a six-electron reduction with the formatoborohydride **28** as an intermediate. Differently from BTM, borane–ammonia complex reduced CO_2_ to the formyl group; conversely, with the borane dimethylamine complex (BDM), the two-electron reduction product **28** reacted with dimethylamine, derived from BDM, leading to amide **30** and blocking further reduction (Scheme 10). In the presence of *^t^*BuOK, methyl borate **29** proved to be an effective methylating agent of nitrile **25,** obtaining the methylated product **26** in 75% yield; in a similar way, two different nucleophiles, such as aniline **31** and thiophenol **33,** were methylated in the same reaction condition.

**Scheme 11:**
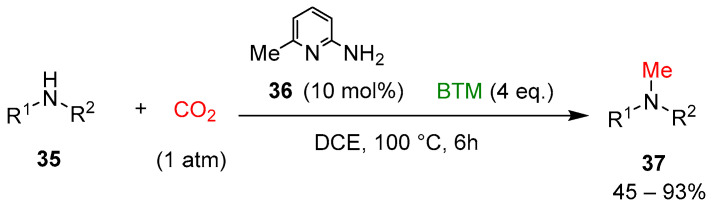
Selective methylation of amines with CO_2_.

**Scheme 12:**
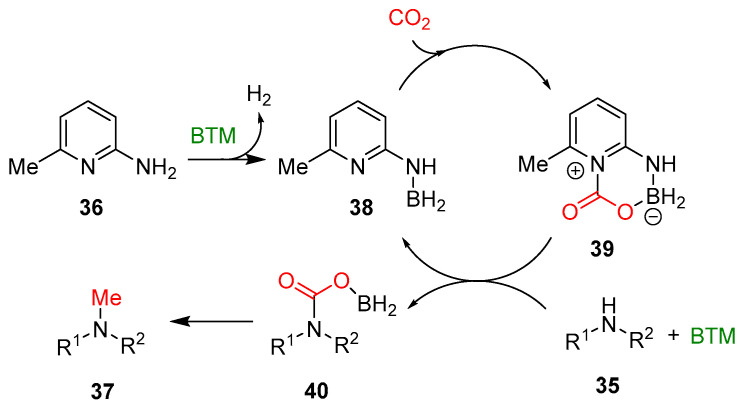
Reaction mechanism of methylation of amines with CO_2_.

Recently, the selective methylation of amines with CO_2_ was examined [23] by combining the organocatalyst 6-amino-2-picoline **36** and BTM (Scheme 11) to form a stable intramolecular frustrated Lewis pairs catalyst (Scheme 12), most of the secondary amines **35** used were *N*-alkyl or *N*-aryl anilines, with only two examples of alkyl heterocyclic amines, affording methylation products **37** in moderate to good yields.

On the basis of a series of control experiments, NMR and high-resolution mass spectrometry (HRMS) analyses, a possible reaction mechanism was proposed (Scheme 12). The first step is the reaction of 6-amino-2-picoline **36** and excess BTM leading to aminoborane **38**, with the quantitative evolution of H_2_, followed by the CO_2_ activation with zwitterionic intermediate **39**. The next steps are less evident, and the authors speculate the formation of intermediate **40** that is further reduced to product **37**.

**Scheme 13:**
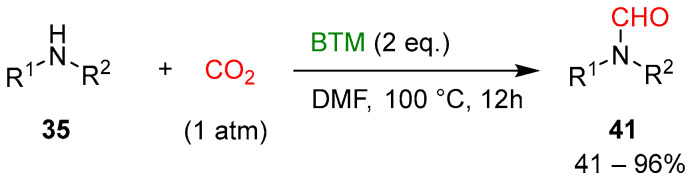
Formylation of amines with CO_2_.

Without a catalyst, halving the equivalents of BTM and with DMF as solvent (Scheme 13), the reduction afforded the corresponding monoformylation products **41**, an intermediate hypothesized in the reduction from **40** to product **37** (Scheme 12); most of the amines **35** used were primary anilines and yields were generally good.

**Scheme 14:**
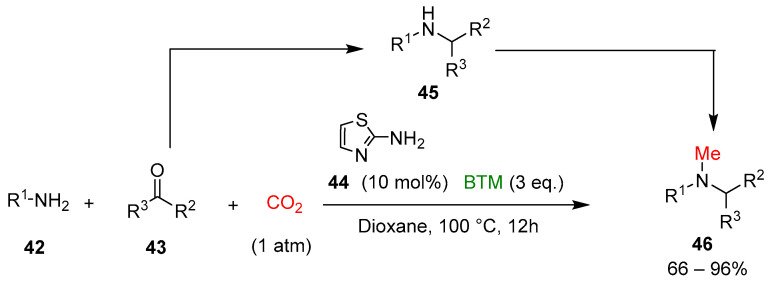
Four-component reductive methylation of primary amines with CO_2_.

Finally, a tandem four-component reductive methylation of primary amines **42** was realized, coupling a reductive amination to secondary amines **45**, with the reduction of CO_2_ to a methyl group, synthesizing tertiary *N*-methylamines **46** [24] (Scheme 14).

**Scheme 15:**
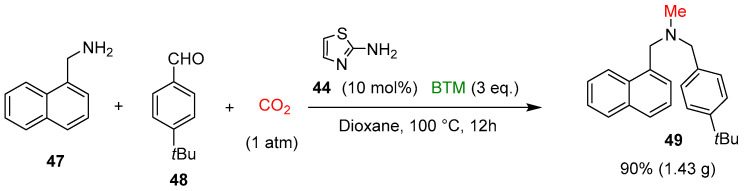
Synthesis of the antifungal agent butenafine **49**.

On the contrary of catalyst **36**, 2-aminothiazole **44** catalyzed the reaction with higher efficiency, whereas BTM was the best choice in the screening of different types of boron-based reducing agents. The study of the scope of the reaction involved a large number of amines **42**, aldehydes and ketones **43,** leading to products in mostly good yields; the gram-scale synthesis of the antifungal agent butenafine **49** was an example of the potentiality of the present synthetic method (Scheme 15).

## 3. Carbon–Nitrogen Double Bond Reduction

### 3.1. Reduction of Hydrazones and Azines

**Scheme 16:**
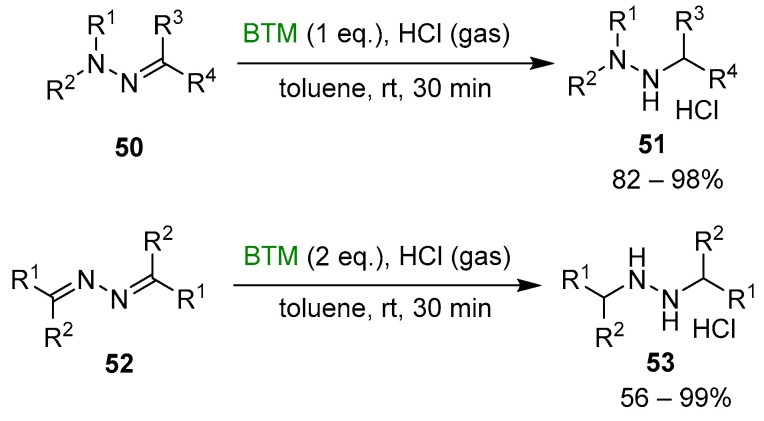
Reduction of hydrazones and azines.

Although the reduction of hydrazones and azines is hampered by opposite conjugation effects, the presence of an acid in the reaction medium can activate the C=N bond to the attack of nucleophiles [25,26]. The improved stability of BTM in a strong acidic medium enabled the efficient reduction of both hydrazones **50** and azines **52** (Scheme 16), affording a wide range of highly functionalized mono-, di- and trialkyl hydrazines as stable and safe hydrochlorides and in excellent yields for most of the compounds.

**Scheme 17:**
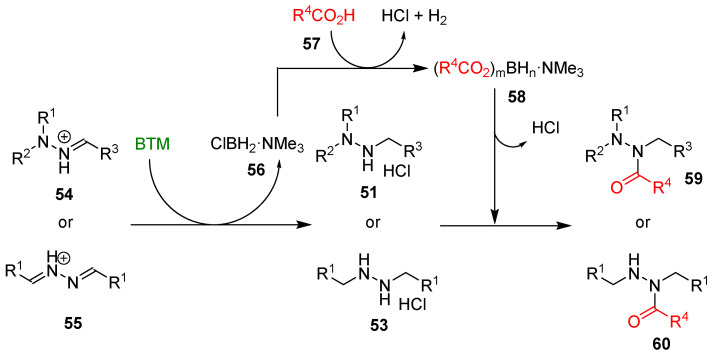
The proposed mechanism of the synthesis of hydrazides.

The work-up operationally simple was another credit of the method, as the byproduct of reduction **56** (Scheme 17) was soluble in toluene, contrary to products **51** or **53,** which are completely insoluble (except for two compounds) and easily separated by filtration.

**Scheme 18:**
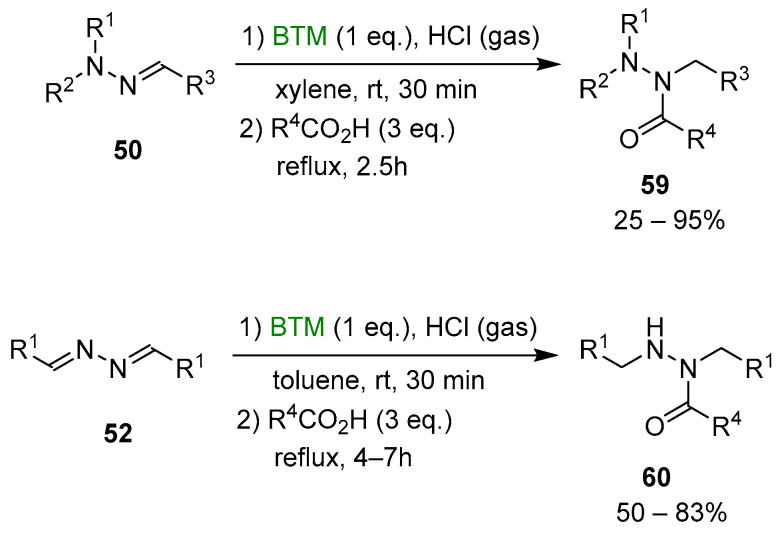
Synthesis of hydrazides.

Byproduct **56** was exploited in the “one pot” synthesis of hydrazides by adding carboxylic acid **57** at the end of the reduction step and producing, in situ, a mixture of acyloxyboranes **58** (Scheme 17) that proved to be an effective acylating agent of both alkylhydrazine hydrochloride **51** and **53** (Scheme 18); the yields were susceptible to the bulkiness of both carboxylic acids and alkylhydrazines limiting the reaction to less hindered hydrazones and azines derived from aldehydes. The tight steric requirement for the acylation by the acyloxyboranes made the synthesis of hydrazides **60** completely selective without the formation of the related diacyl hydrazines side-product, generally observed with common acylating agents.

### 3.2. Reduction of Oximes

**Scheme 19:**
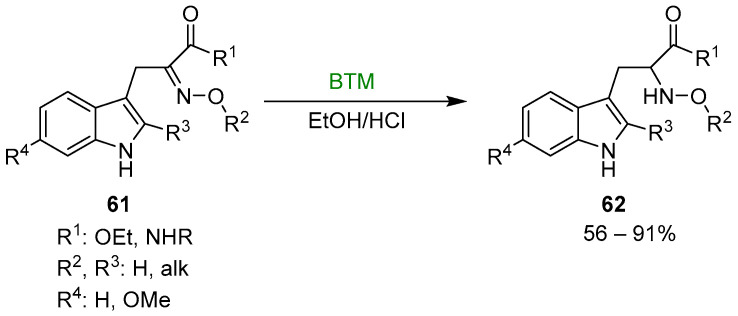
Synthesis of *N*-hydroxy derivatives of tryptophan **62**.

**Scheme 20:**
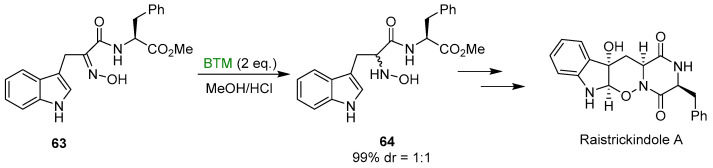
Synthesis of raistrickindole A.

Early studies on the reduction of oximes by BTM and HCl were directed to the synthesis of *N*-hydroxy derivatives of tryptophan **62** (Scheme 19), useful intermediates for the synthesis of β-Carbolines [27,28,29,30], fungal metabolites Neoechinulins [31,32] and recently the marine fungal metabolite raistrickindole A [33] (Scheme 20); with some oximes the reduction of indole to indoline (see Section 3.3) was observed [32].

**Scheme 21:**
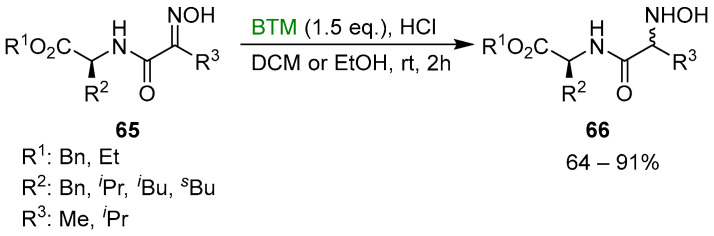
Synthesis of *N*-hydroxy dipeptides.

Subsequently, the synthetic method was extended to the synthesis of a new type of pseudopeptides, the *N*-hydroxy dipeptides **66** [34], as a diastereoisomeric mixture (Scheme 21).

**Scheme 22:**
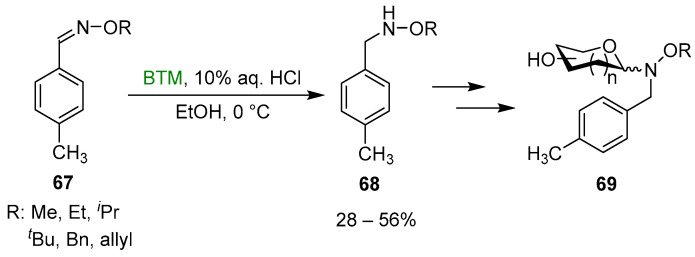
Synthesis of *N*-alkyl-hydroxylamines **68**.

Reduction of oximes was the easiest method of synthesis of the *N*-alkyl-hydroxylamines **68** [35], requisite for the neoglycosylation reaction optimization in the synthesis of glycosylated **69** (Scheme 22); the yields were mediocre to moderate, likely due to the use of diluted HCl aqueous solution.

**Scheme 23:**
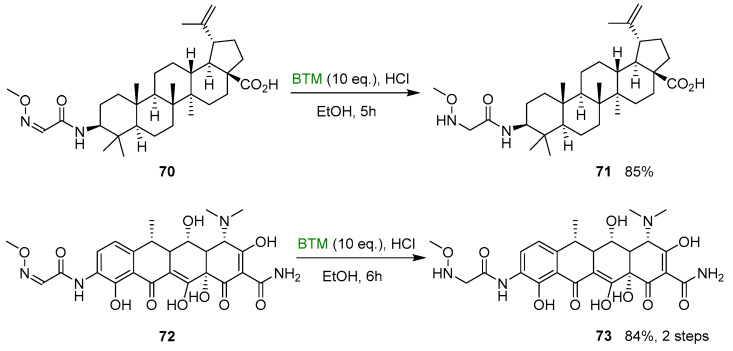
Synthesis of *N*-alkyl-hydroxylamines **71** and **73**.

Similarly, the betulinic derivative **71** [36] and 9-amino doxycycline derivative **73** [37], suitable substrates for the neoglycosylation reaction, were synthesized from the corresponding oximes in good yields, making use of HCl in ethanol and large excess of BTM (Scheme 23).

**Scheme 24:**
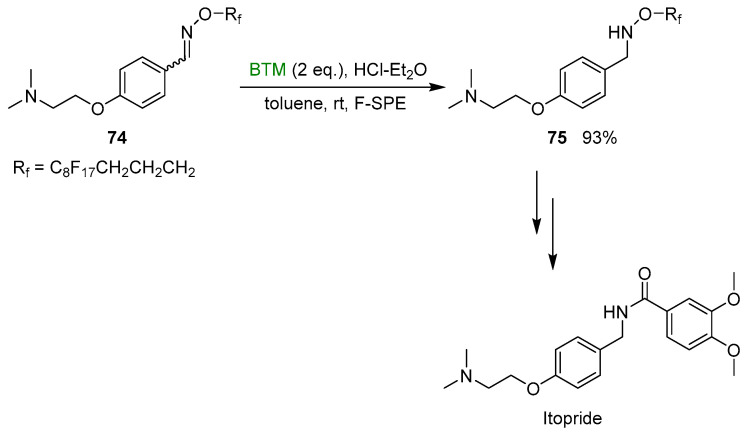
Synthesis of itopride.

Finally, a new fluorous-tagged hydroxylamine [38], as an ammonia equivalent, was successfully exploited in the synthesis of itopride, a drug used for the treatment of functional dyspepsia; the work-up step was simplified by fluorous solid-phase extraction (F-SPE), relative with this strategy (Scheme 24).

### 3.3. Transfer Hydrogenation of Aromatic N-Heterocycles

Several aromatic *N*-heterocycles can react with protic acid or acylating agents, leading to salts with an immonium substructure that can be reduced by BTM similarly to the reduction of imines activated with protic acid [2]; reduction of indoles to indolines was reported in a previous review [2].

**Scheme 25:**
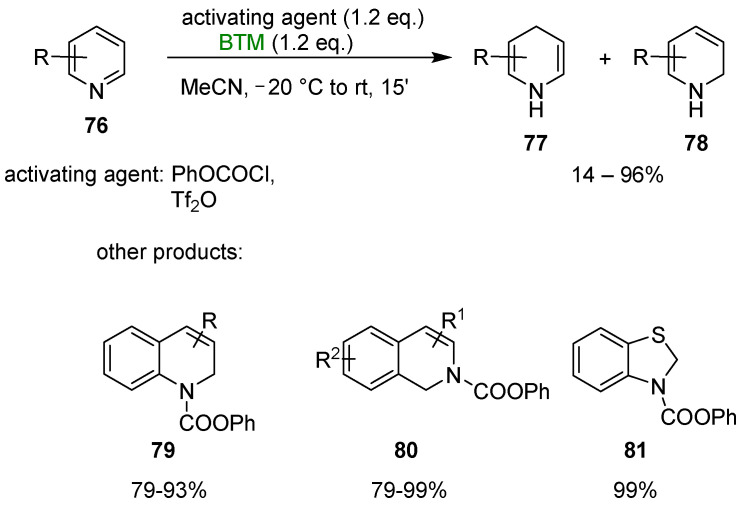
Dearomatization of *N*-heterocycles.

BTM reduced pyridines **76** (Scheme 25), activated by reaction with phenyl chloroformate, to 1,4 dihydropyridine **77** (majority product) and 1,2 dihydropyridine **78** (minority product) [39]; the use of triflic anhydride improved the regioselectivity toward dihydropyridine **77** reaching, in the best case, the 99:1 regioselectivity and 76:24 for the worst case. Substituents in the 4-position completely inverted the selectivity in favor of regioisomer **78**. The reaction could also be applied for the synthesis of other N-heterocycles, such as dihydroquinolines **79**, dihydroisoquinolines **80** and benzothiazoline **81**, among others.

**Scheme 26:**
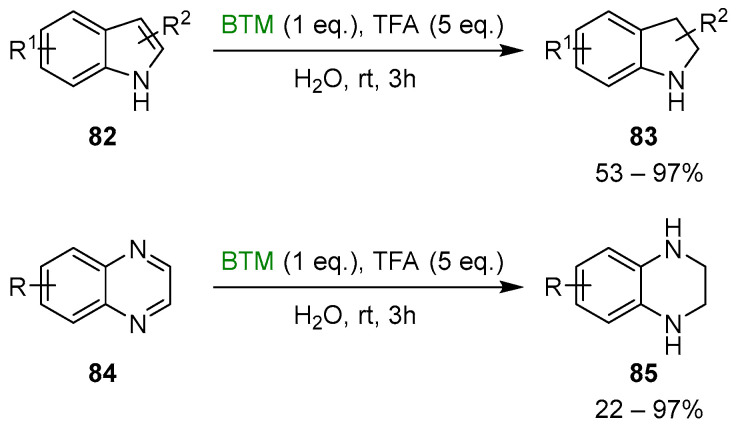
Reduction of indoles **82** and quinoxalines **84**.

Trifluoroacetic acid (TFA), as an acidic activator, offered several opportunities; both indoles **82** and quinoxalines **84** [40] were reduced to indolines **83** and tetrahydroquinoxalines **85,** respectively, in water as solvent (Scheme 26).

**Scheme 27:**
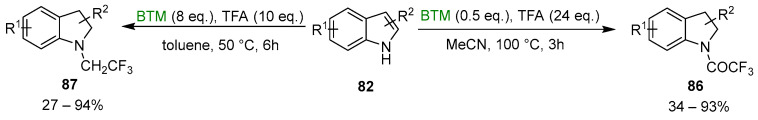
Synthesis of *N*-trifluoroacetylated indolines **86** and *N*-trifluoroethylated indolines **87**.

Tuning the reaction condition and the equivalents of BTM and TFA, as shown in Scheme 27, to go beyond the reduction, obtaining a different product [41]. A reduced amount of BTM, increased equivalents of TFA and the use of an aprotic solvent brought to the *N*-trifluoroacetylated indolines **86**, while the increase of BTM brought to the *N*-trifluoroethylated indolines **87**, similarly to the reduction, by BTM, of carboxylic acid in the presence of amines (Section 2.3, Scheme 8).

**Scheme 28:**
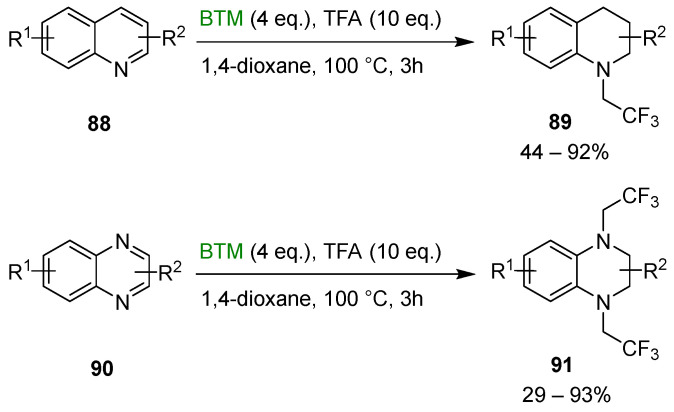
Synthesis of *N*-trifluoroethylated tetrahydroquinoline **89** and *N*-trifluoroethylated tetrahydroquinoxalines **91**.

The synthetic method was successfully extended to the synthesis of *N*-trifluoroethylated tetrahydroquinoline **89** and *N*-trifluoroethylated tetrahydroquinoxalines **91** (Scheme 28) [42].

### 3.4. Selective N-Monomethylation of Primary Anilines

**Scheme 29:**
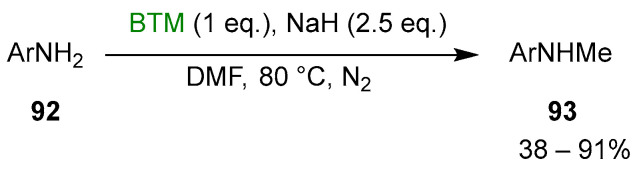
Monomethylation of anilines.

Primary anilines **92** were selectively monomethylated [43] by reaction with BTM and NaH in DMF (Scheme 29).

**Scheme 30:**
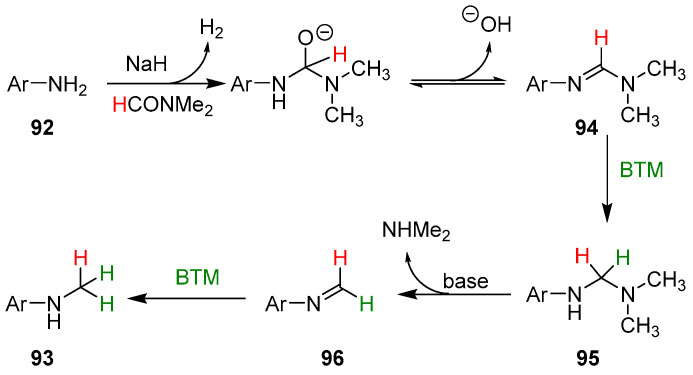
Proposed reaction mechanism of the monomethylation of anilines.

In the proposed reaction mechanism (Scheme 30), the first step is the reaction of aniline **92** with NaH and DMF obtaining amidine **94** that, likewise, the reduction of imines by BTM [2], is reduced to aminal **95**; subsequent elimination of dimethylamine generated an imine that is easily reduced to the final product.

**Scheme 31:**
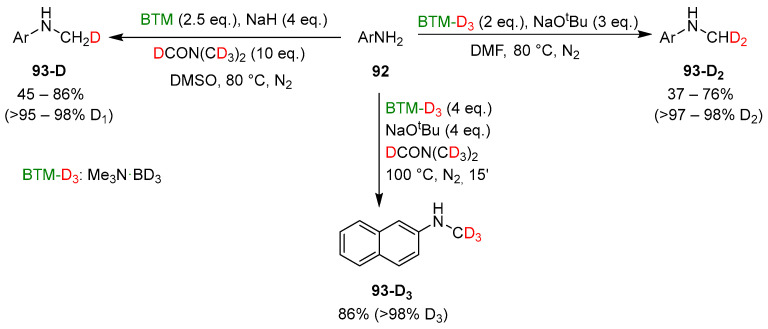
Synthesis of **93-D**, **93-D_2_** and **93-D_3_** anilines.

The sources of the hydrogens of the methyl group in monomethyl anilines **93** (in red and green in Scheme 30) and the easy synthesis of BTM-D_3_ (Me_3_N·BD_3_) by deuterium exchange in acidic D_2_O [2] were exploited in the synthesis of products with specific numbers of deuterium atoms into the methyl groups (Scheme 31) and excellent deuterium incorporation ratio, based on which deuterated reagent was used for the reaction.

## 4. Reduction of Nitrobenzenes to Anilines

**Scheme 32:**
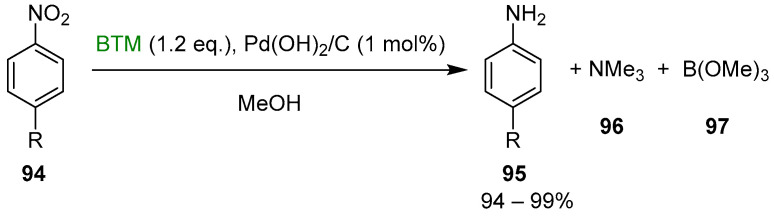
Reduction of nitroaryls **94** to anilines **95**.

BTM is hydrolytically stable in alcohols, but it can be activated in situ through palladium catalysis [44], and the reaction can be coupled with the reduction of nitroaryls **94** to anilines **95** (Scheme 32). Measuring the kinetic of the reaction pointed out that the reduction was faster than hydrogen liberation: BTM acted as hydrogen-transfer reagent and palladium hydride likely as the transient species; indeed, the reaction was an open vessel reduction, even performed at reflux, without concomitant loss of hydrogen.

Credits of the reaction were excellent yield and operationally simple work-up procedure as both byproducts **96** and **97** were removed by simple concentration, and BTM surplus was completely consumed by methanolysis.

**Scheme 33:**
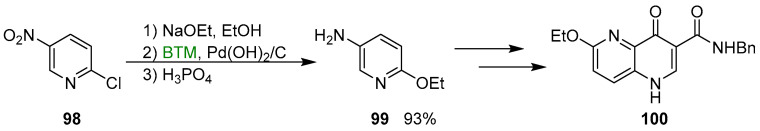
Synthesis of the pyridine **99**.

A practical use of this procedure was the “one pot” multigram synthesis of pyridine **99** (Scheme 33), a key intermediate in the synthesis of quinolone **100**, a subtype-selective GABA-A receptor inverse agonist [45].

## 5. Reductive Deprotection of *N*-Tritylamines

**Scheme 34:**
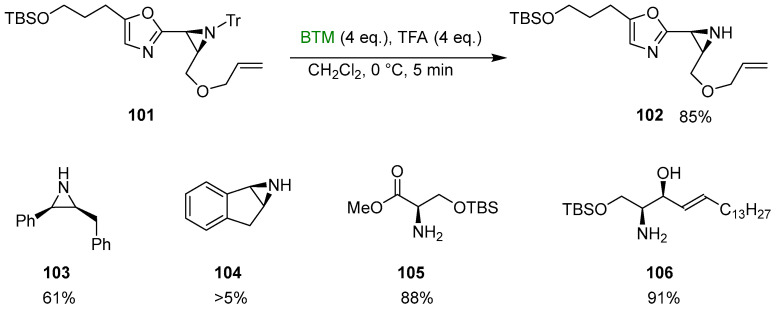
Reductive deprotection of *N*-tritylamines.

BTM reacts with some carbenium ions [11] by hydride abstraction; this reaction was exploited for the trapping of trityl cation in the deprotection of N-tritylamines, especially for sensitive substrates such as *N*-tritylaziridine **101** (Scheme 34) [46,47], an intermediate in the synthesis of aziridinomitosenes.

In addition, the method was tested for the deprotection of the aziridines **103** and **104** [48] with a low yield in the latter case due to problems with aziridine ring opening; in the absence of potential complications, the yields were excellent as for the protected serine **105** [48] and intermediate **106** in the synthesis of phytosphingosines [49].

## 6. Photocatalytic Difluoromethylation of Unactivated Alkenes

**Scheme 35:**
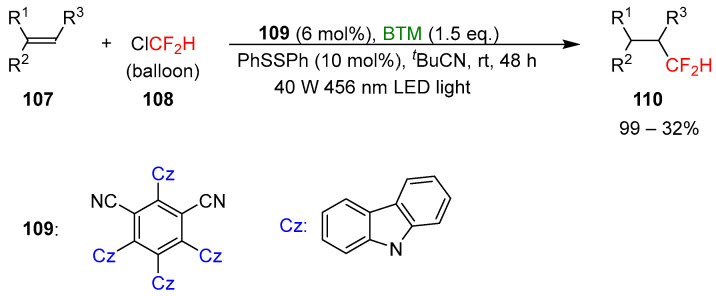
Photocatalytic difluoromethylation of unactivated alkenes.

Ligated boryl radicals, with the general formula L_B_^+^-R_2_B^•−^, are intermediates in the activation of halogenated compounds by a reaction of halogen atom transfer (XAT) owing to the nucleophilic character of boryl radicals. Exploiting this process, BTM was utilized for the photocatalytic trifluoromethylation [50] of unactivated alkenes **107** by activation of Freon-22 **108**, an inexpensive feedstock, under 456 nm blue LED light irradiation (Scheme 35).

**Scheme 36:**
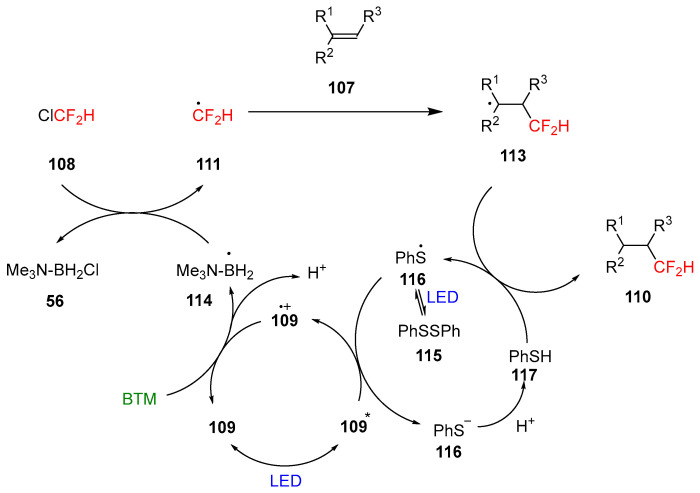
Proposed mechanism of the reaction.

Difluoromethylated product **110** is of enormous interest in pharmaceutical and agrochemical science owing to the properties of the group CF_2_H, bioisostere of hydroxyl, thiol, and amine groups. Alkenes suitable for the reaction protocol were extremely broad with the limitation of sterically encumbered tetra-substituted alkenes, unreactive, styrenes and electron-deficient alkenes where the reduction was a competitive reaction. The tolerance of various functional groups was also proved in the late-stage functionalization of complex pharmaceutical molecules and natural products; the yields were generally good, with limited examples with low yields. Supported by experimental and calculation results, the proposed mechanism of the reaction is shown in Scheme 36.

Aryl thiyl radical **116** is generated from disulfide homolysis under blue light irradiation and subsequently reduced to thiolate **116** by the excited state of **109**; the produced oxidized form **109^.+^** is reduced back by BTM generating the amine-boryl radical **114** that undergoes a XAT reaction with Freon-22 **108** to generate the transient radical **111**. Subsequently, the intermolecular radical addition with the alkene substrate **107** and the quench of the radical adduct **113** by thiol **117** completed the reaction.

## 7. Reductive Cleavage of Acetals

**Scheme 37:**
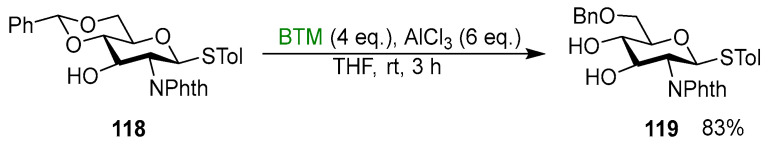
Regioselective cleavage of benzylidene acetal **118.**

Regioselective cleavage of cyclic acetals in the presence of a Lewis acid is the main application of BTM in the field of organic synthesis, and it is almost completely related to carbohydrate chemistry. Carbohydrates protected as 4,6-*O*-benzylidene acetals were regioselectively reduced by BTM in the presence of AlCl_3_ [51,52,53,54,55,56,57,58,59,60,61,62,63,64,65,66,67,68,69,70,71,72,73,74,75,76,77,78,79,80,81,82,83,84,85,86,87,88,89,90,91,92,93,94,95,96,97,98,99,100,101,102], forming a free alcohol at one position and a benzyl ether protection at the other, useful for further modifications; Scheme 37 shows an example [93].

**Scheme 38:**
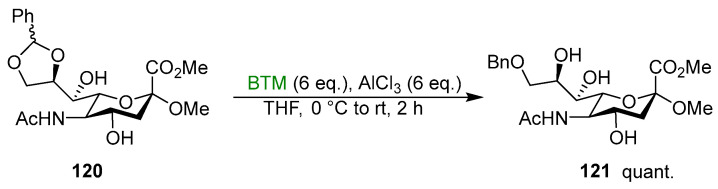
Regioselective cleavage of benzylidene acetal **121.**

Alternative acidic activators were BF_3_·Et_2_O [103,104,105,106,107,108,109,110,111,112,113,114,115,116,117,118], Me_2_BBr [119] and methanesulfonic acid [120]. Five-membered cyclic benzylidene acetals were suitable reactants for the synthetic protocol as well [121,122,123,124,125,126,127,128,129,130,131,132,133,134]; Scheme 38 shows a recent example [134].

**Scheme 39:**
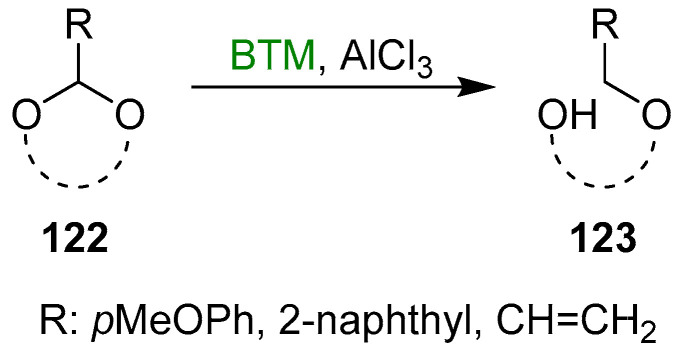
Cleavage of cyclic acetals **122**.

Acetals **122** (Scheme 39) with R different from the phenyl group allowed us to obtain the reduced product **123** with a hydroxyl protected with a different protecting group instead of the benzyl group, allowing more flexibility in the synthesis. Several examples were reported in the literature for R = *p*MeOPh (for six [135,136,137,138] and five [139] membered cyclic acetals), 2-naphthyl (for six- [140,141,142,143,144,145] and five- [146,147,148] membered cyclic acetals) and vinyl (five-membered cyclic acetals [149]).

**Scheme 40:**
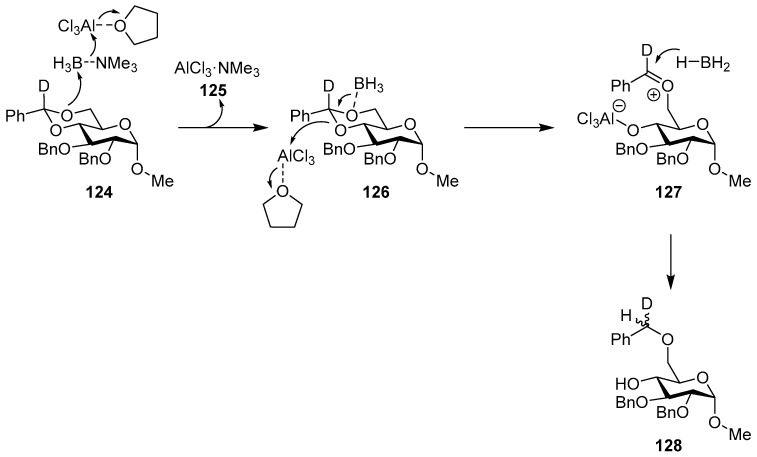
Proposed mechanism for the reaction in THF.

The reaction was extensively investigated, and an early study [150] showed that the addition of two equivalents of water to the reaction mixture (four equivalents of BTM and six equivalents of AlCl_3_) improved the efficiency of benzylidene reductive cleavage without the observation of products of benzylidene acetal hydrolysis and rate enhancement of approximately four times. In order to decipher the mechanistic details of the reaction, several model compounds, kinetic experiments, ^11^B NMR spectroscopy, computational calculations, deuterium labeling, alternative reducing reagents and solvents were used [151,152,153,154], bringing to the proposed mechanism for the reaction in THF, where the Lewis acid is complexed to the solvent (Scheme 40). In the first step, BTM is activated by AlCl_3_, making the borane the most electrophilic species and leading to the interaction with the most electron-rich oxygen in intermediate **126**, whilst the driving force is the formation of the highly stabilized AlCl_3_·NMe_3_ **125**; the opening of the acetal is obtained by the action of a second Lewis acid molecule with the formation of an oxocarbenium ion **127** as the rate-controlling step; finally, oxocarbenium ion is then reduced with low stereoselectivity to give product **128**.

**Scheme 41:**
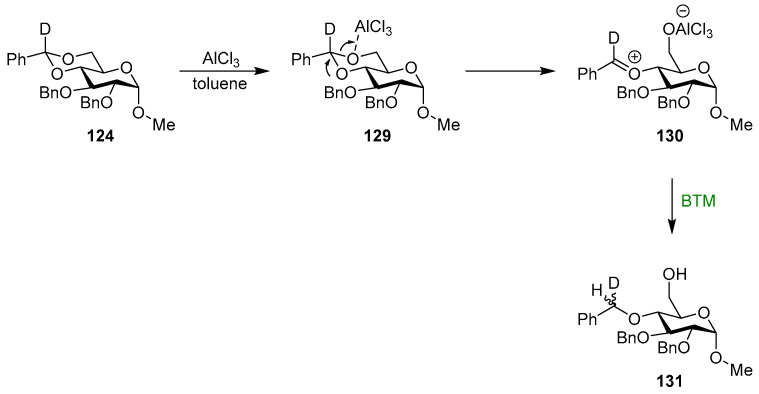
Proposed mechanism for the reaction in toluene.

The reaction in toluene had different regioselectivity and usually gave low yields due to degradation. In this case, in the proposed mechanism (Scheme 41), the strongest Lewis acid is AlCl_3,_ which reacts very fast to give the oxocarbenium ion **130** that is then reduced by BTM, with low stereoselectivity to give product **131**.

## 8. Conclusions

In conclusion, BTM has several credits as a reagent in modern organic synthesis. It is relatively inexpensive, and considering its low molecular weight, it has a low price per mole. It is a stable solid with a good safety profile linked with its relative inertness. Its reactivity can be opportunely activated in the reaction medium, generally in the presence of Lewis or Brønsted acids. BTM undergoes rapid deuterium exchange in acidic D_2_O, allowing easy conversion to BTM-D_3_, an effective reagent for the synthesis of deuterium-labeled compounds. BTM is very soluble in a wide variety of solvents, offering more versatility in the reaction options. The tolerance of various functional groups was a well-substantiated feature of this reagent. The main application of BTM is the regioselective cleavage of cyclic acetals, a reaction of great importance for carbohydrate chemistry. Carbon–nitrogen double bond reduction is another class of reactions where the activation by acids plays an important role in the BTM reactivity. Finally, recent findings in organocatalysis have contributed to develop some innovative applications of BTM, such as the CO_2_ utilization as feedstock and the radical chemistry by photocatalysis.

## Data Availability

Not applicable.

## References

[1] Lane C.F. (1973). The borane.amine complexes. Aldrichimica Acta.

[2] Hutchins R.O., Learn K., Nazer B., Pytlewski D., Pelter A. (1984). Amine boranes as selective reducing and hydroborating agents. A review. Org. Prep. Proced. Int..

[3] Carboni B., Monnier B. (1999). Recent developments in the chemistry of amine- and phosphine-boranes. Tetrahedron.

[4] Matos K., Pichlmair S., Burkhardt E.R. (2007). Boron reagents for reductive amination. Chim. Oggi.

[5] Matos K., Burkhardt E.R., Shioiri T., Izawa K., Konoike T. (2010). Direct reductive amination with amine boranes. Pharmaceutical Process Chemistry.

[6] Sumerin V., Chernichenko K., Schulz F., Lesleka M., Rieger B., Repo T., Erker G., Douglas W.S. (2012). Amine-borane mediated metal-free hydrogen activation and catalytic hydrogenation. Frustrated Lewis Pairs I.

[7] Lau S., Gasperini D., Webster R.L. (2021). Amine–boranes as transfer hydrogenation and hydrogenation reagents: A mechanistic perspective». Angew. Chem. Int. Ed..

[8] Faverio C., Boselli M.F., Medici F., Benaglia M. (2020). Ammonia borane as a reducing agent in organic synthesis. Bioinorg. Chem. Appl..

[9] Karthikeyan S., Sedlak R., Hobza P. (2011). On the nature of stabilization in weak, medium, and strong charge-transfer complexes: CCSD(T)/CBS and SAPT calculations. J. Phys. Chem. A.

[10] Lo R., Manna D., Lamanec M., Dračínský M., Bouř P., Wu T., Bastien G., Kaleta J., Miriyala M.V., VŠpirko V. (2022). The stability of covalent dative bond significantly increases with increasing solvent polarity. Nat. Commun..

[11] Funke M.-A., Mayr H. (1997). Kinetics and mechanism of the reactions of amine boranes with carbenium ions. Chem. Eur. J..

[12] Gohzu S., Tada M. (1986). Regioselective reduction of polyketones on silica gel surface with borane–trimethylamine complex. Chem. Lett..

[13] Sarko C.R., Guch I.C., DiMare M. (1994). Chelation-controlled protocol for the diastereoselective reduction of ketones. J. Org. Chem..

[14] Zhang Y., Ye Q., Ponomareva L.V., Cao Y., Liu Y., Cui Z., Van Lanen S.G., Voss S.R., She Q.-B., Thorson J.S. (2019). Total synthesis of griseusins and elucidation of the griseusin mechanism of action. Chem. Sci..

[15] Le Corre M., Gheerbrant E., Le Deit H. (1989). Trimethylamine–borane bromide as alternative reagent for reductive bromation of aromatic carbonyl compounds. J. Chem. Soc. Chem. Commun..

[16] Malmquist J., Ström P. (2012). Multiple labeling of a potent CX_3_CR1 antagonist for the treatment of multiple sclerosis. J. Label. Compd. Radiopharm..

[17] Stengel I., Götz G., Weil M., Bäuerle P. (2015). A dinuclear (Bpy)Pt ^II^ -decorated crownophane. Eur. J. Org. Chem..

[18] Trapani G., Reho A., Latrofa A., Liso G. (1990). Trimethylamine-borane a useful reagent in the one-pot preparation of carboxylic esters from carboxylic acids. Synthesis.

[19] Trapani G., Reho A., Latrofa A. (1983). Trimethylamine-borane as useful reagent in the N-acylation or N-alkylation of amines by carboxylic acids. Synthesis.

[20] Ménard G., Stephan D.W. (2013). CO_2_ reduction via aluminum complexes of ammonia boranes. Dalton Trans..

[21] Roy L., Ghosh B., Paul A. (2017). Lewis acid promoted hydrogenation of CO_2_ and HCOO—By amine boranes: Mechanistic insight from a computational approach. J. Phys. Chem. A.

[22] Zhang X., Wang S., Xi C. (2019). α-Methylation of 2-arylacetonitrile by a trimethylamine-borane-CO_2_ system. J. Org. Chem..

[23] Zhang Y., Zhang H., Gao K. (2021). Borane–trimethylamine complex as a reducing agent for selective methylation and formylation of amines with CO_2_. Org. Lett..

[24] Xiong F., Cheng Q., Dang Y., Gao K. (2022). A tandem reduction of primary amines, carbonyl compounds, CO_2_, and boranes catalyzed by in situ formed frustrated lewis pairs. Org. Chem. Front..

[25] Perdicchia D., Licandro E., Maiorana S., Baldoli C., Giannini C. (2003). A new ‘one-pot’ synthesis of hydrazides by reduction of hydrazones. Tetrahedron.

[26] Perdicchia D. (2023). Ionic hydrogenation of azines: An efficient synthesis of 1,2-dialkylhydrazines. Tetrahedron.

[27] Plate R., Hermkens P.H.H., Smits J.M.M., Ottenheijm H.C.G. (1986). Nitrone cycloaddition in the stereoselective synthesis of β-carbolines from *N*-hydroxytryptophan. J. Org. Chem..

[28] Plate R., Hermkens P.H.H., Smits J.M.M., Nivard R.J.F., Ottenheijm H.C.G. (1987). Employment of nitriles in the stereoselective cycloaddition to nitrones. J. Org. Chem..

[29] Hermkens P.H.H., Van Maarseveen J.H., Berens H.W., Smits J.M.M., Kruse C.G., Scheeren H.W. (1990). Intramolecular Pictet-Spengler reaction of *N*-alkoxytryptophans and tryptamines. 2. Synthesis of corynanthe alkaloid derivatives containing a tetrahydro-1,2-oxazine as the D ring. J. Org. Chem..

[30] Hermkens P.H.H., Van Maarseveen J.H., Cobben P.L.H.M., Ottenheijm H.C.G., Kruse C.G., Scheeren H.W. (1990). Syntheses of 1,3-disubstituted *N*-oxy-β-carbolines by the Pictet-Spengler reactions of *N*-oxy-tryptophan and -tryptamine derivatives. Tetrahedron.

[31] Plate R., Ottenheijm H.C.G. (1986). Synthesis of 2-(dimethylallyl)-*N*-hydroxytryptophans from indole. Tetrahedron Lett..

[32] Plate R., Nivard R.J.F., Ottenheijm H.C.G. (1987). Conversion of *N*-hydroxytryptophans into α,β-dehydrotryptophan. An approach to the neoechinulin series. J. Chem. Soc. Perkin Trans. 1.

[33] Pham T.L., Sae-Lao P., Toh H.H.M., Csókás D., Bates R.W. (2022). The total synthesis of Raistrickindole A. J. Org. Chem..

[34] Lawrence J., Cointeaux L., Maire P., Vallée Y., Blandin V. (2006). *N*-Hydroxy and *N*-acyloxy peptides: Synthesis and chemical modifications. Org. Biomol. Chem..

[35] Langenhan J.M., Endo M.M., Engle J.M., Fukumoto L.L., Rogalsky D.R., Slevin L.K., Fay L.R., Lucker R.W., Rohlfing J.R., Smith K.R. (2011). Synthesis and biological evaluation of RON-neoglycosides as tumor cytotoxins. Carbohydr. Res..

[36] Goff R.D., Thorson J.S. (2009). Enhancing the divergent activities of betulinic acid via neoglycosylation. Org. Lett..

[37] Zhang J., Ponomareva L.V., Marchillo K., Zhou M., Andes D.R., Thorson J.S. (2013). The synthesis and antibacterial activity of doxycycline neoglycosides. J. Nat. Prod..

[38] Nielsen S.D., Smith G., Begtrup M., Kristensen J.L. (2010). Synthesis and application of a new fluorous-tagged ammonia equivalent. Chem. Eur. J..

[39] Heusler A., Fliege J., Wagener T., Glorius F. (2021). Substituted dihydropyridine synthesis by dearomatization of pyridines. Angew. Chem. Int. Ed..

[40] Zeng Y.-F., Li Y.-N., Zhou M.-X., Han S., Guo Y., Wang Z. (2022). Metal-free hydrogenation of *N*-heterocycles with trimethylamine borane and TFA in aqueous solution. Adv. Synth. Catal..

[41] Zeng Y.-F., Zhou M.-X., Li Y.-N., Wu X., Guo Y., Wang Z. (2022). Switchable reductive *N*-trifluoroethylation and *N*-trifluoroacetylation of indoles with trifluoroacetic acid and trimethylamine borane. Org. Lett..

[42] Li Y.-N., Zhou M.-X., Wu J.-B., Wang Z., Zeng Y.-F. (2022). Tandem reduction and trifluoroethylation of quinolines and quinoxalines with trifluoroacetic acid and trimethylamine borane. Org. Biomol. Chem..

[43] Meng J., Xia H.M., Xu A.-Q., Wang Y.-F., Wang Z., Zhang F.-L. (2020). Selective *N*-monomethylation of primary anilines with the controllable installation of *N*-CH_2_D, *N*-CHD_2_, and *N*-CD_3_ units. Org. Biomol. Chem..

[44] Couturier M., Tucker J.L., Andresen B.M., Dubé P., Brenek S.J., Negri J.T. (2001). Palladium catalyzed activation of borane–amine adducts: Rate enhancement of amine–borane methanolysis in the reduction of nitrobenzenes to anilines. Tetrahedron Lett..

[45] Beaudin J., Bourassa D.E., Bowles P., Castaldi M.J., Clay R., Couturier M.A., Karrick G., Makowski T.W., McDermott R.E., Meltz C.N. (2003). Synthesis and purification of 6-ethoxy-4-oxo-1,4-dihydro-[1,5]naphthyridine-3-carboxylic acid benzylamide. Org Process Res Dev.

[46] Klapars E.A.V., Naidu B.N., Piotrowski D.W., Tucci F.C. (2000). Enantiocontrolled synthesis of (1*S*,2*S*)-6-desmethyl-(methylaziridino)mitosene. J. Am. Chem. Soc..

[47] Vedejs E., Naidu B.N., Klapars A., Warner D.L., Li V.-S., Na Y., Kohn H. (2003). Synthetic enantiopure aziridinomitosenes: Preparation, reactivity, and DNA alkylation studies. J. Am. Chem. Soc..

[48] Vedejs E., Klapars A., Warner D.L., Weiss A.H. (2001). Reductive deprotection of *N*-tritylaziridines. J. Org. Chem..

[49] Park J.-J., Lee J.H., Li Q., Diaz K., Chang Y.-T., Chung S.-K. (2008). Divergent syntheses of all stereoisomers of phytosphingosine and their use in the construction of a ceramide library. Bioorg. Chem..

[50] Zhang Z.-Q., Sang Y.-Q., Wang C.-Q., Dai P., Xue X.-S., Piper J.L., Peng Z.-H., Ma J.-A., Zhang F.-G., Wu J. (2022). Difluoromethylation of unactivated alkenes using freon-22 through tertiary amine-borane-triggered halogen atom transfer. J. Am. Chem. Soc..

[51] Ek M., Garegg P.J., Hultberg H., Oscarson S. (1983). Reductive ring openings of carbohydrate benzylidene acetals using borane-trimethylamine and aluminium chloride. Regioselectivity and solvent dependance. J. Carbohydr. Chem..

[52] Fügedi P., Birberg W., Garegg P.J., Pilotti A. (1987). Syntheses of a branched heptasaccharide having phytoalexin-elicitor activity. Carbohydr. Res..

[53] Sato S., Nunomura S., Nakano T., Ito Y., Ogawa T. (1988). An efficient approach to stereoselective glycosylation of ceramide derivatives: Use of pivaloyl group as a stereocontrolling auxiliary. Tetrahedron Lett..

[54] Garegg P.J. (1992). Saccharides of biological importance: Challenges and opportunities for organic synthesis. Acc. Chem. Res..

[55] Garegg P.J., Olsson L., Oscarson S. (1995). Synthesis of methyl (ethyl 2-*O*-acyl-3,4-di-*O*-benzyl-1-thio-*β*-*D*-glucopyranosid)uronates and evaluation of their use as reactive *β*-selective glucuronic acid donors. J. Org. Chem..

[56] Karst N., Jacquinet J.-C. (2000). Chemical synthesis of *β*-D-Glc*p*A(2SO_4_)-(1→3)-D-Gal*p*NAc(6SO_4_), the disaccharide repeating unit of shark cartilage chondroitin sulfate D, and of its methyl *β*-D-glycoside derivative. J. Chem. Soc. Perkin Trans. 1.

[57] Svansson L., Johnston B.D., Gu J.-H., Patrick B., Pinto B.M. (2000). Synthesis and conformational analysis of a sulfonium-ion analogue of the glycosidase inhibitor castanospermine. J. Am. Chem. Soc..

[58] Li X., Ohtake H., Takahashi H., Ikegami S. (2001). Direct methylenation of partially benzyl-protected sugar lactones by dimethyltitanocene. Synlett.

[59] Hoffmann B., Zanini D., Ripoche I., Bürli R., Vasella A. (2001). Oligosaccharide analogues of polysaccharides, part 22, synthesis of cyclodextrin analogues containing a buta-1,3-diyne-1,4-diyl or a butane-1,4-diyl unit. Helv. Chim. Acta.

[60] Sherman A.A., Yudina O.N., Mironov Y.V., Sukhova E.V., Shashkov A.S., Menshov V.M., Nifantiev N.E. (2001). Study of glycosylation with *N*-trichloroacetyl-D-glucosamine derivatives in the syntheses of the spacer-armed pentasaccharides sialyl lacto-*N*-neotetraose and sialyl lacto-*N*-tetraose, their fragments, and analogues. Carbohydr. Res..

[61] Kitov P.I., Bundle D.R. (2001). Synthesis and structure–activity relationships of di- and trisaccharide inhibitors for Shiga-like toxin type 1. J. Chem. Soc. Perkin Trans. 1.

[62] Sherman A.A., Yudina O.N., Shashkov A.S., Menshov V.M., Nifant’ev N.E. (2001). Synthesis of Neu5Ac- and Neu5Gc-*α*-(2→6′)-lactosamine 3-aminopropyl glycosides. Carbohydr. Res..

[63] Amaya T., Tanaka H., Yamaguchi T., Naoto Shibuya N., Takahashi T. (2001). The first synthesis of tetraglucosyl glucitol having phytoalexin-elicitor activity in rice cells based on a sequential glycosylation strategy. Tetrahedron Lett..

[64] Chowdhury A.R., Siriwardena A., Boons G.-J. (2002). A highly convergent approach for the synthesis of disaccharide repeating units of peptidoglycan. Tetrahedron Lett..

[65] Elsayed G.A., Zhu T., Boons G.-J. (2002). Demixing libraries of saccharides using a multi-linker approach in combination with a soluble polymeric support. Tetrahedron Lett..

[66] Dohi H., Nishida Y., Furuta Y., Uzawa H., Yokoyama S.-I., Ito S., Mori H., Kobayashi K. (2002). Molecular design and biological potential of galacto-type trehalose as a nonnatural ligand of Shiga toxins. Org. Lett..

[67] Manabe S., Ito Y. (2002). On-resin real-time reaction monitoring of solid-phase oligosaccharide synthesis. J. Am. Chem. Soc..

[68] Takahashi T., Okano A., Amaya T., Tanaka H., Doi T. (2002). Solid-Phase Synthesis of a phytoalexin elicitor-active tetraglucosyl glucitol. Synlett.

[69] Kanemitsu T., Wong C.-H., Kanie O. (2002). Solid-phase synthesis of oligosaccharides and on-resin quantitative monitoring using gated decoupling ^13^C NMR. J. Am. Chem. Soc..

[70] Ágoston K., Kerékgyártó J., Hajkó J., Batta G., Lefeber D.J., Kamerling J.P., Vliegenthart J.F.G. (2002). Synthesis of fragments of the glycocalyx glycan of the *Parasiteschistosoma mansoni*. Chem. Eur. J..

[71] Tanaka H., Amaya T., Takahashi T. (2003). Parallel synthesis of multi-branched oligosaccharides related to elicitor active pentasaccharide in rice cell based on orthogonal deprotection and glycosylation strategy. Tetrahedron Lett..

[72] Alpe M., Oscarson S., Svahnberg P. (2003). Synthesis of *Cryptococcus neoformans* capsular polysaccharide structures. IV. Construction of thioglycoside donor blocks and their subsequent assembly. J. Carbohydr. Chem..

[73] Tarling C.A., Withers S.G. (2004). The synthesis of a series of modified mannotrisaccharides as probes of the enzymes involved in the early stages of mammalian complex *N*-glycan formation. Carbohydr. Res..

[74] Tsvetkov Y.E., Nifantiev N.E. (2005). Enhanced sialylating activity of *O*-chloroacetylated 2-thioethyl sialosides. Synlett.

[75] Wang J., Li J., Chen H.-N., Chang H., Tanifum C.T., Liu H.-H., Czyryca P.G., Chang C.-W.T. (2005). Glycodiversification for the optimization of the Kanamycin class aminoglycosides. J. Med. Chem..

[76] Veselý J., Rohlenová A., Džoganová M., Trnka T., Tišlerová I., Šaman D., Ledvina M. (2006). Preparation of ethyl 2-azido-2-deoxy-1-thio-*β*-*D*-mannopyranosides, and their rearrangement to 2-*S*-ethyl-2-thio-β-*D*-mannopyranosylamines. Synthesis.

[77] Khatuntseva E.A., Tsvetkov Y.E., Grachev A.A., Nifant’ev N.E. (2005). Synthesis of aminoethyl glycosides of type 2 chain A tetrasaccharide and related trisaccharides. Russ. J. Org. Chem..

[78] Matsuoka K., Goshu Y., Takezawa Y., Mori T., Sakamoto J.-I., Yamada A., Onaga T., Koyama T., Hatano K., Snyder P.W. (2007). Practical synthesis of fully protected globotriaose and its glycopolymers. Carbohydr. Polym..

[79] Yamada A., Hatano K., Matsuoka K., Koyama T., Esumi Y., Koshino H., Hino K., Nishikawa K., Natori Y., Terunuma D. (2006). Syntheses and Vero toxin-binding activities of carbosilane dendrimers periphery-functionalized with galabiose. Tetrahedron.

[80] Komarova B.S., Tsvetkov Y.E., Knirel Y.A., Zähringer U., Pier G.B., Nifantiev N.E. (2006). Synthesis of a common trisaccharide fragment of glycoforms of the outer core region of the *Pseudomonas aeruginosa* lipopolysaccharide. Tetrahedron Lett..

[81] Nakano J., Akihiro Ishiwata A., Ohta H., Ito Y. (2007). Synthesis of complex-type glycans derived from parasitic helminths. Carbohydr. Res..

[82] Sukhova E.V., Dubrovskii A.V., Tsvetkov Y.E., Nifantiev N.E. (2007). Synthesis of oligosaccharides related to the HNK-1 antigen. 5. Synthesis of a sulfo-mimetic of the HNK-1 antigenic trisaccharide. Russ. Chem. Bull..

[83] Tatai J., Osztrovszky G., Kajtár-Peredy M., Fügedi P. (2008). An efficient synthesis of *L*-idose and *L*-iduronic acid thioglycosides and their use for the synthesis of heparin oligosaccharides. Carbohydr. Res..

[84] Komarova B.S., Tsvetkov Y.E., Pier G.B., Nifantiev N.E. (2008). First Synthesis of pentasaccharide glycoform I of the outer core region of the *Pseudomonas aeruginosa* lipopolysaccharide. J. Org. Chem..

[85] Lin Y.-S., Tungpradit R., Sinchaikul S., An F.-M., Liu D.-Z., Phutrakul S., Chen S.-T. (2008). Targeting the delivery of glycan-based paclitaxel prodrugs to cancer cells via glucose transporters. J. Med. Chem..

[86] Schwardt O., Gäthje H., Vedani A., Mesch S., Gao G.-P., Spreafico M., von Orelli J., Kelm S., Ernst B.J. (2009). Examination of the Biological Role of the α (2→ 6)-Linked Sialic Acid in Gangliosides Binding to the Myelin-Associated Glycoprotein (MAG). Med. Chem..

[87] Rasmussen T.S., Jensen H.H. (2011). Chiral pool synthesis of calystegine A3 from 2-deoxyglucose via a Brown allylation. Carbohydr. Res..

[88] Titz A., Marra A., Cutting B., Smieško M., Papandreou G., Dondoni A., Ernst B. (2012). Conformational constraints: Nature does it best with sialyl Lewis^x^. Eur. J. Org. Chem..

[89] Xia L., Zheng R.B., Lowary T.L. (2012). Revisiting the specificity of an *α*-(1→4)-mannosyltransferase involved in mycobacterial methylmannose polysaccharide biosynthesis. ChemBioChem.

[90] Tamura J.-I., Tsutsumishita-Nakai N., Nakao Y., Kawano M., Kato S., Takeda N., Nadanaka S., Kitagawa H. (2012). Synthesis and interaction with Midkine of biotinylated chondroitin sulfate tetrasaccharides. Bioorg. Med. Chem. Lett..

[91] Takeda N., Ikeda-Matsumi R., Ebara-Nagahara K., Otaki-Nanjo M., Taniguchi-Morita K., Nanjo M., Tamura J.-I. (2012). Synthesis of heparan sulfate tetrasaccharide as a substrate for human heparanase. Carbohydr. Res..

[92] Komarova B.S., Tsvetkov Y.E., Pier G.P., Nifantiev N.E. (2012). Synthesis of pentasaccharides corresponding to the glycoform II of the outer core region of the *Pseudomonas aeruginosa* lipopolysaccharide. Carbohydr. Res..

[93] Zhang J., Zou L., Lowary T.L. (2013). Synthesis of the tolerance-inducing oligosaccharide lacto-*N*-fucopentaose III bearing an activated linker. ChemistryOpen.

[94] Kitamura Y., Koshino H., Nakamura T., Tsuchida A., Nitoda T., Kanzaki H., Matsuoka K., Takahashi S. (2013). Total synthesis of the proposed structure for pochonicine and determination of its absolute configuration. Tetrahedron Lett..

[95] Yudina O.N., Tsvetkov Y.E., Nifantiev N.E. (2015). Synthesis of 2-aminoethyl glycosides of chitooligosaccharides. Russ. Chem. Bull..

[96] Fan Q.-H., Pickens J.B., Striegler S., Gervaise C.D. (2016). Illuminating the binding interactions of galactonoamidines during the inhibition of *β*-galactosidase (*E. coli*). Bioorg. Med. Chem..

[97] d’Ortoli T.A., Widmalm G. (2016). Synthesis of the tetrasaccharide glycoside moiety of Solaradixine and rapid NMR-based structure verification using the program CASPER. Tetrahedron.

[98] Vinnitskiy D.Z., Ustyuzhanina N.E., Andrey S.D., Shashkov A.S., Nifantiev N.E. (2017). Synthesis and NMR analysis of model compounds related to fucosylated chondroitin sulfates: GalNAc and Fuc(1 → 6)GalNAc derivatives. Carbohydr. Res..

[99] Tsvetkov Y.E., Yashunsky D.V., Sukhova E.V., Kurbatova E.A., Nifantiev N.E. (2017). Synthesis of oligosaccharides structurally related to fragments of *Streptococcus pneumoniae* type 3 capsular polysaccharide. Russ. Chem. Bull..

[100] Demeter F., Veres F., Herczeg M., Borbás A. (2018). Short synthesis of Idraparinux by applying a 2-*O*-Methyl-4,6-*O*-arylmethylene thioidoside as a 1,2-trans-*α*-selective glycosyl donor. Eur. J. Org. Chem..

[101] Tateda N., Ajisaka K., Ishiguro M., Miyazaki T. (2019). Synthesis of 5a,5a′-dicarba-*D*-glucobioses from conformationally restricted carbaglucosyl triflates using SN2-type inversion with carbaglucosyl nucleophiles. Bioorg. Med. Chem..

[102] Dhaene S., Van Der Eycken J., Beerens K., Franceus J., Desmet T., Caroen J. (2020). Synthesis, trehalase hydrolytic resistance and inhibition properties of 4- and 6-substituted trehalose derivatives. J. Enzyme Inhib. Med. Chem..

[103] Inamura S., Fukase K., Kusumoto S. (2001). Synthetic study of peptidoglycan partial structures. synthesis of tetrasaccharide and octasaccharide fragments. Tetrahedron Lett..

[104] Hesek D., Lee M., Morio K.-I., Mobashery S. (2004). Synthesis of a fragment of bacterial cell wall. J. Org. Chem..

[105] Inamura S., Fujimoto Y., Kawasaki A., Shiokawa Z., Woelk E., Heine H., Lindner B., Inohara N., Kusumoto S., Fukase K. (2006). Synthesis of peptidoglycan fragments and evaluation of their biological activity. Org. Biomol. Chem..

[106] Bohn M.L., Colombo M.I., Stortz C.A., Rúveda E.A. (2006). A comparative study of the influence of some protecting groups on the reactivity of *D*-glucosamine acceptors with a galactofuranosyl donor. Carbohydr. Res..

[107] Bohn M.L., Colombo M.I., Pisano P.L., Stortz C.A., Rúveda E.A. (2007). Differential *O*-3/*O*-4 regioselectivity in the glycosylation of *α* and *β* anomers of 6-*O*-substituted *N*-dimethylmaleoyl-protected *D*-glucosamine acceptors. Carbohydr. Res..

[108] Fujimoto Y., Konishi Y., Kubo O., Hasegawa M., Inohara N., Fukase K. (2009). Synthesis of crosslinked peptidoglycan fragments for investigation of their immunobiological functions. Tetrahedron Lett..

[109] Danieli E., Lay L., Proietti D., Berti F., Costantino P., Adamo R. (2011). First synthesis of *C. difficile* PS-II cell wall polysaccharide repeating unit. Org. Lett..

[110] Colombo M.I., Rúveda E.A., Stortz C.A. (2011). Regioselectivity of the glycosylation of *N*-dimethylmaleoyl-protected hexosamine acceptors. An experimental and DFT approach. Org. Biomol. Chem..

[111] Adamo R., Romano M.R., Berti F., Leuzzi R., Tontini M., Danieli E., Cappelletti E., Cakici O.S., Swennen E., Pinto V. (2012). Phosphorylation of the synthetic hexasaccharide repeating unit is essential for the induction of antibodies to *Clostridium difficile* PSII cell wall polysaccharide. ACS Chem. Biol..

[112] Wang N., Huang C.-Y., Hasegawa M., Inohara N., Fujimoto Y., Fukase K. (2013). Glycan sequence-dependent Nod2 activation investigated by using a chemically synthesized bacterial peptidoglycan fragment library. ChemBioChem.

[113] Adamo R., Micoli F., Proietti D., Berti F. (2014). Efficient synthesis of Meningococcal X polysaccharide repeating unit (*N*-acetylglucosamine-4-phosphate) as analytical standard for polysaccharide determination. Synth. Commun..

[114] Enugala R., Pires M.J.D., Marques M.M.B. (2014). Synthesis of the NAG–NAM disaccharide via a versatile intermediate. Carbohydr. Res..

[115] Wang Q., Matsuo Y., Pradipta A.R., Inohara N., Fujimoto Y., Fukase K. (2016). Synthesis of characteristic mycobacterium peptidoglycan (PGN) fragments utilizing with chemoenzymatic preparation of meso-diaminopimelic acid (DAP), and their modulation of innate immune responses. Org. Biomol. Chem..

[116] Wang N., Hasegawa H., Huang C.-Y., Fukase K., Fujimoto Y. (2017). Synthesis of peptidoglycan fragments from *Enterococcus faecalis* with Fmoc-strategy for glycan elongation. Chem. Asian J..

[117] Del Bino L., Calloni I., Oldrini D., Raso M.M., Cuffaro R., Ardá A., Codée J.D.C., Jiménez-Barbero J., Adamo R. (2019). Regioselective glycosylation strategies for the synthesis of group Ia and Ib *Streptococcus* related glycans enable elucidating unique conformations of the capsular polysaccharides. Chem. Eur. J..

[118] Dallabernardina P., Benazzi V., Laman J.D., Seeberger P.H., Loeffler F.F. (2021). Automated glycan assembly of peptidoglycan backbone fragments. Org. Biomol. Chem..

[119] Ghosh M., Dulina R.G., Kakarla R., Sofia M.J. (2000). Efficient synthesis of a stereochemically defined carbohydrate scaffold: Carboxymethyl 2-acetamido-6-azido-4-*O*-benzyl-2-deoxy-*α*-*D*-glucopyranoside. J. Org. Chem..

[120] Périon R., Lemée L., Ferrières V., Duval R., Plusquellec D. (2003). A new synthesis of the oligosaccharide domain of acarbose. Carbohydr. Res..

[121] Ando H., Koike Y., Koizumi S., Ishida H., Kiso M. (2005). 1,5-Lactamized sialyl acceptors for various disialoside syntheses: Novel method for the synthesis of glycan portions of Hp-s6 and HLG-2 gangliosides. Angew. Chem. Int. Ed..

[122] Ando H., Shimizu H., Katano Y., Koike Y., Koizumi S., Ishida H., Kiso M. (2006). Studies on the *α*-(1→4)- and *α*-(1→8)-fucosylation of sialic acid for the total assembly of the glycan portions of complex HPG-series gangliosides». Carbohydr. Res..

[123] Tanaka H., Nishiura Y., Adachi M., Takahashi T. (2006). Synthetic study of *α*(2,8) oligosialoside using *N*-Troc sialyl *N*-phenyltrifluoroimidate. Heterocycles.

[124] Shelke S.V., Gao G.-P., Mesch S., Gäthje H., Kelm S., Schwardt O., Ernst B. (2007). Synthesis of sialic acid derivatives as ligands for the myelin-associated glycoprotein (MAG). Bioorg. Med. Chem..

[125] Tanaka H., Nishiura Y., Takahashi T. (2009). Stereoselective synthesis of *α*(2,9) di- to tetrasialic acids, using a 5,4-*N*,*O*-carbonyl protected thiosialoside. J. Org. Chem..

[126] Hanashima S., Ishikawa D., Akai S., Sato K.-I. (2009). Synthesis of the starfish ganglioside LLG-3 tetrasaccharide. Carbohydr. Res..

[127] Meinke S., Schroven A., Thiem J. (2011). Sialic acid C-glycosides with aromatic residues: Investigating enzyme binding and inhibition of *Trypanosoma Cruzi* trans-sialidase. Org. Biomol. Chem..

[128] Shimizu H., Iwayama Y., Imamura A., Ando H., Ishida H., Kiso M. (2011). Synthesis of the disialic acid-embedded glycan part of ganglioside HPG-1. Biosci. Biotechnol. Biochem..

[129] Schumann B., Pragani R., Anish C., Pereira C.L., Seeberger P.H. (2014). Synthesis of conjugation-ready zwitterionic oligosaccharides by chemoselective thioglycoside activation. Chem. Sci..

[130] Cheallaigh N.A., Oscarson S. (2016). Synthesis of building blocks for an iterative approach towards oligomers of the *Streptococcus Pneumoniae* type 1 zwitterionic capsular polysaccharide repeating unit. Can. J. Chem..

[131] Podvalnyy N.M., Malysheva N.N., Panova M.V., Zinin A.I., Chizhov A.O., Orlova A.V., Kononov L.O. (2017). Stereoselective sialylation with *O*-trifluoroacetylated thiosialosides: Hydrogen bonding involved?. Carbohydr. Res..

[132] Shirasaki J., Tanaka H.-N., Konishi M., Hirose Y., Imamura A., Ishida H., Kiso M., Ando H. (2020). Systematic strategy utilizing 1,5-lactamization for the synthesis of the trisialylated galactose unit of c-series gangliosides. Tetrahedron Lett..

[133] Wu Y.-F., Tsai Y.-F., Huang Y.-S., Shih J.-F. (2020). Total synthesis of the echinodermatous ganglioside LLG-3 possessing the biological function of promoting the neurite outgrowth. Org. Lett..

[134] Johansson E., Caraballo R., Elofsson M. (2021). Synthesis of 4-*O*-alkylated *N*-acetylneuraminic acid derivatives. J. Org. Chem..

[135] Hirschmann R., Ducry L., Smith A.B. (2000). Development of an efficient, regio- and stereoselective route to libraries based on the *β*-*D*-glucose scaffold. J. Org. Chem..

[136] Morii Y., Matsuda H., Ohara K., Hashimoto M., Miyairi K., Okuno T. (2005). Synthetic studies on oligosaccharides composed of 5-thioglucopyranose units. Bioorg. Med. Chem..

[137] Danieli E., Lalot J., Murphy P.V. (2007). Selective protecting group manipulations on the 1-Deoxynojirimycin scaffold. Tetrahedron.

[138] Noguchi S., Takemoto S., Kidokoro S.-I., Yamamoto K., Hashimoto M. (2011). Syntheses of cellotriose and cellotetraose analogues as transition state mimics for mechanistic studies of cellulases. Bioorg. Med. Chem..

[139] Weïwer M., Chen C.-C., Kemp M.M., Linhardt R.J. (2009). Synthesis and biological evaluation of non-hydrolyzable 1,2,3-triazole-linked sialic acid derivatives as neuraminidase inhibitors. Eur. J. Org. Chem..

[140] Borbás A., Szabó Z.B., Szilágyi L., Bényei A., Lipták A. (2002). Dioxane-type (2-naphthyl)methylene acetals of glycosides and their hydrogenolytic transformation into 6-*O*- and 4-*O*-(2-naphthyl)methyl (NAP) ethers. Tetrahedron.

[141] Tanaka H., Tateno Y., Takahashi T. (2012). Convergent stereoselective synthesis of multiple sulfated GlcN*α*(1,4)GlcA*β*(1,4) dodecasaccharides. Org. Biomol. Chem..

[142] Oka H., Koyama T., Hatano K., Matsuoka K. (2012). Synthetic studies of bi-fluorescence-labeled maltooligosaccharides as substrates for *α*-amylase on the basis of fluorescence resonance energy transfer (FRET). Bioorg. Med. Chem..

[143] Takeda N., Tamura J.-I. (2014). Synthesis of biotinylated keratan sulfate repeating disaccharides. Biosci. Biotechnol. Biochem..

[144] Takeda-Okuda N., Yamaguchi Y., Uzawa J., Tamura J.-I. (2017). Synthesis of a biotinylated keratan sulfate tetrasaccharide composed of dimeric Gal*β*1-4GlcNAc6S*β*. Carbohydr. Res..

[145] Herczeg M., Demeter F., Balogh T., Kelemen V., Borbás A. (2018). Rapid synthesis of*L*-idosyl glycosyl donors from *α*-thioglucosides for the preparation of heparin disaccharides. Eur. J. Org. Chem..

[146] Lipták A., Borbás A., Jánossy L., Szilágyi L. (2000). Preparation of (2-naphthyl)methylene acetals of glycosides and their hydrogenolytic transformation into 2-naphthylmethyl (NAP) ethers. Tetrahedron Lett..

[147] Borbás A., Szabó Z.B., Szilágyi L., Bényei A., Lipták A. (2002). Stereoselective (2-naphthyl)methylation of sugar hydroxyls by the hydrogenolysis of diastereoisomeric dioxolane-type (2-naphthyl)methylene acetals. Carbohydr. Res..

[148] Aoyagi T., Ohira S., Fuse S., Uzawa J., Yamaguchi Y., Tanaka H. (2016). The *α*-glycosidation of partially unprotected *N*-acetyl and *N*-glycolyl sialyl donors in the absence of a nitrile solvent effect. Chem. Eur. J..

[149] Matsushita K., Sato Y., Funamoto S., Tamura J.-I. (2014). Side reactions with 2,2,2-trichloroethoxysulfates during the synthesis of glycans. Carbohydr. Res..

[150] Sherman A.A., Mironov Y.V., Yudina O.N., Nifantiev N.E. (2003). The presence of water improves reductive openings of benzylidene acetals with trimethylaminoborane and aluminium chloride. Carbohydr. Res..

[151] Johnsson R., Mani K., Cheng F., Ellervik U. (2006). Regioselective reductive openings of acetals; mechanistic details and synthesis of fluorescently labeled compounds. J. Org. Chem..

[152] Johnsson R., Olsson D., Ellervik U. (2008). Reductive openings of acetals: Explanation of regioselectivity in borane reductions by mechanistic studies. J. Org. Chem..

[153] Johnsson R., Cukalevski R., Dragén F., Ivanisevic D., Johansson I., Petersson L., Wettergren E.E., Yam K.B., Yang B., Ellervik U. (2008). Reductive openings of benzylidene acetals. kinetic studies of borane and alane activation by lewis acids. Carbohydr. Res..

[154] Johnsson R., Ohlin M., Ellervik U. (2010). Reductive openings of benzylidene acetals revisited: A mechanistic scheme for regio- and stereoselectivity. J. Org. Chem..

